# New automated analysis to monitor neutrophil function point-of-care in the intensive care unit after trauma

**DOI:** 10.1186/s40635-020-0299-1

**Published:** 2020-03-14

**Authors:** Lillian Hesselink, Roy Spijkerman, Emma de Fraiture, Suzanne Bongers, Karlijn J. P. Van Wessem, Nienke Vrisekoop, Leo Koenderman, Luke P. H. Leenen, Falco Hietbrink

**Affiliations:** 1 Department of Trauma Surgery, University Medical Center Utrecht, Heidelberglaan 100, 3584 CX Utrecht, the Netherlands; 2 Department of Respiratory Medicine, Wilhelmina Children’s Hospital, Lundlaan 6, 3584 EA Utrecht, the Netherlands; 3 Center for Translational Immunology, University Medical Center Utrecht, Heidelberglaan 100, 3584 CX Utrecht, the Netherlands; 4 Department of Trauma Surgery, University Medical Center Utrecht, Heidelberglaan 100, 3584 CX Utrecht, the Netherlands

**Keywords:** Intensive care, Trauma, Infections, Neutrophils, Point-of-care, Bedside, Monitoring

## Abstract

**Background:**

Patients often develop infectious complications after severe trauma. No biomarkers exist that enable early identification of patients who are at risk. Neutrophils are important immune cells that combat these infections by phagocytosis and killing of pathogens. Analysis of neutrophil function used to be laborious and was therefore not applicable in routine diagnostics. Hence, we developed a quick and point-of-care method to assess a critical part of neutrophil function, neutrophil phagosomal acidification. The aim of this study was to investigate whether this method was able to analyze neutrophil functionality in severely injured patients and whether a relation with the development of infectious complications was present.

**Results:**

Fifteen severely injured patients (median ISS of 33) were included, of whom 6 developed an infection between day 4 and day 9 after trauma. The injury severity score did not significantly differ between patients who developed an infection and patients who did not (*p* = 0.529). Patients who developed an infection showed increased acidification immediately after trauma (*p* = 0.006) and after 3 days (*p* = 0.026) and a decrease in the days thereafter to levels in the lower normal range. In contrast, patients who did not develop infectious complications showed high-normal acidification within the first days and increased tasset to identify patients at risk for infections after trauma and to monitor the inflammatory state of these trauma patients.

**Conclusion:**

Neutrophil function can be measured in the ICU setting by rapid point-of-care analysis of phagosomal acidification. This analysis differed between trauma patients who developed infectious complications and trauma patients who did not. Therefore, this assay might prove a valuable asset to identify patients at risk for infections after trauma and to monitor the inflammatory state of these trauma patients.

**Trial registration:**

Central Committee on Research Involving Human Subjects, NL43279.041.13. Registered 14 February 2014. https://www.toetsingonline.nl/to/ccmo_search.nsf/Searchform?OpenForm.

## Background

Trauma patients are prone to develop infectious complications. The risk of these infections is mainly determined by the severity of the injury and the following dysregulation of the immune response [1, 2]. Over half of the severely injured patients admitted to the intensive care unit (ICU) develop an infection during hospitalization, generally after 5 days post-trauma [3]. Although infection-related mortality rates decreased over the past decades [4], severe infections such as sepsis remain a substantial cause of morbidity and mortality after trauma worldwide [5, 6].

Until now, it has not been possible to recognize patients who will develop these relatively late complications in an early phase after trauma. Generally used biomarkers such as leukocyte counts and C-reactive protein (CRP) become positive during infections and therefore have limited prognostic value [7]. Since neutrophils are the first responders to both tissue damage and invading pathogens [8, 9], multiple studies focused on neutrophils as potential biomarkers [10–16]. Biomarkers that were suggested after trauma included neutrophil C5aR expression [13, 14], neutrophil extracellular traps (NETs) [17], neutrophil CD64 expression [18, 19], neutrophil cell size [20], and neutrophil formyl-methionyl-leucyl-phenylalanine (fMLF)-induced FcγRII expression, of which only the latter was found to be an early marker in multiple trauma cohorts [21, 22]. Neutrophil fMLF-induced FcγRII expression measured immediately after trauma showed high sensitivity (90%) for the prediction of severe sepsis ≥ 5 days post-trauma [21]. This suggests that those patients who are at risk for severe infectious complications can be identified on admission already. However, specificity was rather low (20%) [21], indicating that a large portion of these high-risk patients did not develop septic shock eventually. Possibly, this is because of decisions made in the days after admission. For example, the type of chosen antibiotics and timing of surgery can influence the risk of infections [23–25]. Therefore, there is an unmet need for a biomarker to monitor high-risk trauma patients in the early days after admission.

During these initial days, an adequately functioning neutrophil compartment is critical to prevent infectious complications as these cells are vital in the uptake and destruction of microbes [1, 8, 9]. Neutrophil phagocytosis and neutrophil phagosomal acidification are two critical steps in this process [26]. Both can be assessed by flow analysis of neutrophils phagocytosing bioparticles coupled to pH-sensitive and pH-insensitive dyes [27]. Neutrophil subsets, including CD16^dim^/CD62L^bright^ cells (banded neutrophils) that were found to have different antimicrobial function [28], can be analyzed in the same assay with the same machine as for determination of neutrophil function. Until now, however, flow analysis was too complex, operator-dependent, time-consuming, and laborious to be applicable in a clinical setting [28, 29]. Recently, a flow cytometer became available that is able to prepare and analyze whole blood fast (< 25 min), highly reproducible, and fully automated [30–32]. Hence, we used such an approach to develop a new assay for neutrophil function (phagocytosis and phagosomal acidification) and neutrophil subsets that can be performed as a point-of-care test by any health care worker. The aim of this proof-of-principle study was to investigate whether this assay was suitable for monitoring neutrophil functional capacity in the ICU and identify patients who will develop infectious complications after severe trauma.

## Material and methods

### Study design

This prospective cohort study analyzed neutrophil functionality within the first 2 weeks after trauma in severely injured trauma patients. Patients were only included after written informed consent of the patient or his/her legal representative, in accordance with the Declaration of Helsinki. All experiments were performed in accordance with the relevant guidelines and regulations. The study was approved by the University Medical Centre Utrecht ethical review committee (protocol no. 13/325). The trial was registered online on the website of the Central Committee on Research Involving Human Subjects before participant enrollment started (NL43279.041.13). The process and storage of data were in accordance with privacy and ethics regulations.

### Patients

Severely injured patients ≥ 18 years of age with an expected ICU stay of ≥ 48 h were included between November 2018 and July 2019. Patients were excluded if they recently (< 3 months before hospital admission) used immunosuppressive medication, had an immunosuppressive disorder, or were admitted to ICU because of isolated neurologic injury. Blood was drawn in sodium heparine tubes as soon as possible after trauma (< 12 h), after 3 days, 6 days, 10 days, and 15 days. Control blood samples were provided by anonymous, sex- and age-matched, healthy volunteers. Data concerning patient characteristics, trauma mechanism, injuries, resuscitation, and treatment were obtained from the electronic medical record system. The injury severity score (ISS) [33] based on the abbreviated injury scale 2008 (AIS08) [34] was obtained from the National Trauma Registration database that collects data of all trauma patients admitted to the emergency department [35, 36].

### Experimental setup

#### Fluorescent double-labeling of bioparticles

pHrodo® Green *Staphylococcus aureus* (*S. aureus*) BioParticles^TM^ (Thermo Fisher Scientific, Waltham, MA, USA) were labeled with PromoFluor 520 LSS NHS ester (PF520) (PromoCell, Heidelberg, Germany) following the instructions of the manufacturer. In short, PF520 was dissolved in DMSO at a concentration of 2.5 mg/ml and the bioparticles were suspended in a 0.1-M NaHCO_3_ pH 9 buffer at room temperature at a concentration of 10 mg/ml (3 × 10^9^ particles/ml). Bioparticles were sonicated to prevent clumping. Then, PF520 was added drop-wise while the bioparticle suspension was vortexed. The suspension was mixed for 1 h in the dark at room temperature, after which the double-labeled bioparticles were washed 3 times. Bioparticles were suspended in a pH 7.4 buffer containing 20 mM HEPES, 132 mM NaCl, 6 mM KCl, 1 mM MgSO_4_, 1.2 mM KH_2_PO_4_, 1.0 mM CaCl_2_, 5 mM glucose, and 5 mg/ml human serum albumin (Albuman 200 g/l, Sanquin, Amsterdam, the Netherlands).

#### Flow cytometry analysis

All experiments were performed with whole blood using the fully automated AQUIOS CL® “Load & Go” flow cytometer (Beckman Coulter, Brea, CA, USA) at 30 °C [32]. Firstly, the AQUIOS CL® automatically incubated whole blood with the double-labeled *S. aureus* bioparticles (end concentration of 10 × 10^6^/ml) and with antibody-fluorochrome conjugates for the neutrophil receptors CD16 (clone 3G8, PE labeled; Beckman Coulter) and CD62L (clone DREG56, ECD labeled; Beckman Coulter). Then, after 10, 20, 40, and 60 min of incubation, the AQUIOS CL® was programmed to aspirate part of the sample, to lyse red blood cells (RBCs), and to perform flow cytometric analysis of the leukocytes. Lysing is performed by the addition of 335 μl of lysing reagent A (Beckman Coulter) followed by 100 μl of lysing reagent B (Beckman Coulter). Lysing reagent A is a cyanide-free lytic reagent that lyses red blood cells, and lysing reagent B slows the reaction caused by reagent A and preserves the white blood cells for measurement in the flow cell. The .LMD files were exported and analyzed using Kaluza Analysis Software v2.1 (Beckman Coulter).

#### Analysis of neutrophil subsets and functionality

The gating strategy is shown in Supplementary Figures 1 and 2. Granulocytes were identified based on their specific forward/side scatter pattern (Supplementary Figure 1a). Neutrophils were identified by selecting granulocytes with CD16 expression (thereby excluding eosinophils) (Supplementary Figure 1b). Percentages of CD16^dim^/CD62L^bright^ neutrophils, CD16^bright^/CD62L^bright^ neutrophils, and CD16^bright/^CD62L^dim^ neutrophils were analyzed as previously described (Supplementary Figure 2) [37]. The acidification of neutrophil phagolysomes was investigated by analyzing both pHrodo® Green fluorescence and PF520 fluorescence. The fluorescence of pHrodo® Green increases when the pH in the phagolysosome decreases [27, 38], while the fluorescence of PF520 is not sensitive for pH changes. Combined analysis of these fluorochromes allows for assessment of phagocytosis, expressed as percentage of PF520-positive neutrophils, and neutrophil phagosomal acidification, expressed as the ratio pHrodo® Green fluorescence divided by PF520 fluorescence (Supplementary Figure 1c-d). This ratio was measured per PF520-positive neutrophil to correct for the number of phagocytosed bioparticles within the cell. The mean ratio of all neutrophils was used as an indicator of acidification. Additionally, mean fluorescence intensities (MFIs) of PF520 and pHrodo® were calculated to gain insight into changes in MFI over time and how this influences the ratio pHrodo® Green fluorescence divided by PF520 fluorescence.

### Statistical analysis

Data were analyzed with IBM SPSS version 23 (IBM Corporation, NY, USA) and GraphPad Prism version 8 (GraphPad, La Jolla, CA, USA). The distribution of continuous variables was assessed with the use of the Shapiro-Wilk test and through visual inspection. Clinical outcomes and demographics were presented as median with interquartile range (IQR) and compared between outcome groups using a Fisher’s exact test or a Mann-Whitney *U* test, as indicated. Generalized estimating equations (GEE) were used to compare neutrophil subset percentages, neutrophil functionality, PF520 MFI, and pHrodo® MFI over time between patients who later develop an infection and patients who do not, and to correct for within-subject correlation. Outcome data of GEE analysis were presented as the beta coefficient (β) with *p* value. Additionally, to investigate the differences between these groups for every time point, a Mann-Whitney *U* test was used because data were not normally distributed. Furthermore, neutrophil phagosomal acidification after 60 min was compared between the 3 neutrophil subsets using a one-way ANOVA with a follow-up comparison of the means using Tukey’s correction for multiple comparisons, since data were normally distributed. Statistical significance was defined as a *p* value < 0.05.

## Results

### Baseline characteristics

In total, 15 severely injured patients were included. These patients had a median age of 39.0 (24.0–62.0) and median ISS of 33.0 (22.0–36.0) (Table 1). Mechanisms of injuries were traffic accident (*n* = 11), fall from height (*n* = 1), physical abuse (*n* = 1), and gunshot injuries (*n* = 2). Six patients developed infectious complications, all between day 4 and day 9 after trauma. Four patients died, of whom 2 died due to severe traumatic brain injuries, 1 due to infectious complications in combination with pre-existing liver disease, and 1 due to acute intestinal ischemia. No statistically significant differences in baseline and outcome characteristics were found between patients who developed infectious complications and patients who did not.

**Table 1:** Baseline and outcome characteristics

	All patients (*n* = 15)	No infectious complications (*n* = 9)	Infectious complications (*n* = 6)	*P* value
Gender (male/female)	10/5	5/4	5/1	0.580
Age	39.0 (24.0–62.0)	39.0 (27.5–54.0)	40.0 (19.8–68.8)	1.000
ISS	33.0 (22.0–36.0)	29.0 (19.5–38.5)	33.0 (27.8–38.0)	0.529
Resuscitation < 24 h				
FFP	6 (0.0–10.0)	4 (0.0–9.5)	9 (0.0–18.0)	0.46
RBCs	2 (0.0–9.0)	1 (0.0–7.5)	6 (1.5–14.3)	0.27
PLTs	0 (0.0–6.0)	0 (0.0–4.5)	1.5 (0.0–9.8)	0.46
Mechanism of injury				0.275
Traffic	11 (73.3%)	7 (77.8%)	4 (66.7%)	
Fall from height	1 (6.7%)	1 (11.1%)	0	
Physical abuse	1 (6.7%)	1 (11.1%)	0	
Gunshot injury	2 (13.3%)	0	2 (33.3%)	
Open fracture				0.341
Gustilo grade II	1 (6.7%)	1 (11%)	0	
Gustilo grade IIIA	1 (6.7%)	0	1 (16.7%)	
Gustilo grade IIIB	1 (6.7%)	0	1 (16.7%)	
Open wounds				0.379
Minor laceration	4 (26.7%)	2 (22.2%)	2 (33.3%)	
Major laceration	1 (6.7%)	0	1 (16.7%)	
Length of hospital stay (days)	17.0 (12.0–26.0)	15.0 (10.0–24.0)	25.5 (15.3–28.3)	0.145
In hospital mortality	4 (26.7%)	2 (22.2%)	2 (33.3%)	
Causes of death				1.000
Infectious complication	1 (6.7%)	0	1 (16.7%)	
Intestinal ischemia	1 (6.7%)	0	1 (16.7%)	
Traumatic brain injury	2 (13.3%)	2 (22.2%)	0	

Data are presented as median (IQR) or *n* (%). Minor lacerations were defined as lacerations involving cutaneous tissue only. Major lacerations were defined as lacerations involving cutaneous tissue as well as deeper tissues. Variables are compared between patients with infectious complications and patients without infectious complications with Fisher’s exact test or Mann-Whitney *U* test

*ISS* injury severity score, *FFP* fresh frozen plasma, *RBCs* packed red blood cells, *PLTs* platelets

### Neutrophil subsets

**Fig. 1:**
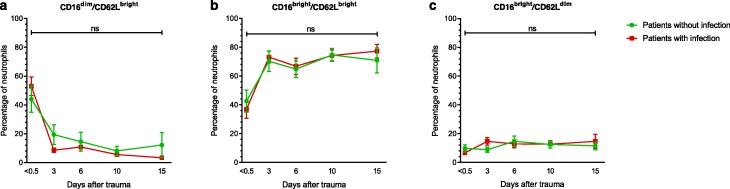
Neutrophil subsets. Percentage of CD16^dim^/CD62L^bright^ cells (**a**), CD16^bright^/CD62L^bright^ cells (**b**), and CD16^bright^/CD62L^dim^ cells (**c**) of patients who developed an infection (red square) and patients who did not (green circle). Patients who developed an infection were compared to patients without infection using generalized estimating equations. No statistically significant differences were found between these groups. Additionally, groups were compared per time point using Student’s *T* test with correction for multiple comparisons with the Hom-Sidak method. Again, no statistically significant differences were found between groups. Data are presented as mean with the standard error of the mean

No statistically significant differences were found in CD16^dim^/CD62L^bright^ neutrophils (*β* = 1.070, *p* = 0.132), CD16^bright^/CD62L^bright^ neutrophils (*β* = − 0.934, *p* = 0.230), and CD16^bright/^CD62L^dim^ (*β* = −0.263, *p* = 0.397) neutrophils over time between patients who developed an infection and patients who did not (Fig. 1). Also, no statistically significant differences were found between these groups when comparing the percentages of these subsets per single time point.

### Neutrophil functionality

**Fig. 2:**
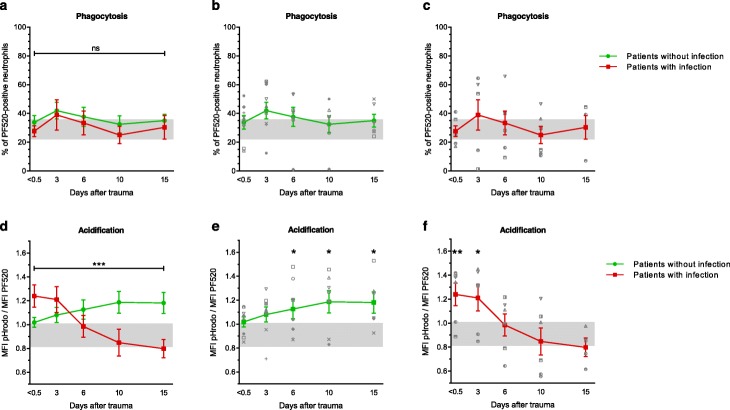
Neutrophil functionality. Neutrophil phagocytosis (**a**–**c**) and neutrophil phagosomal acidification (**d**–**f**) in patients who developed an infection (red square) and patients who did not develop an infection (green circle) after 60 min of incubation with *S. aureus* bioparticles. Patients developed these infections between day 4 and day 9. Patients with infections were compared to patients without infections with generalized estimating equations (Fig. 2a, d). Additionally, patients were compared to healthy control values using a Mann-Whitney *U* test (Fig. 2b, c and Fig. 2e, f). Data are presented as mean with the standard error of the mean. Gray areas represent healthy control values (95% confidence interval). PF520 = PromoFluor 520 LSS. *S. Aureus = Staphylococcus aureus.* Ns = non-significant. *p < 0.05, **p < 0.01, ***p < 0.001

**Fig. 3:**
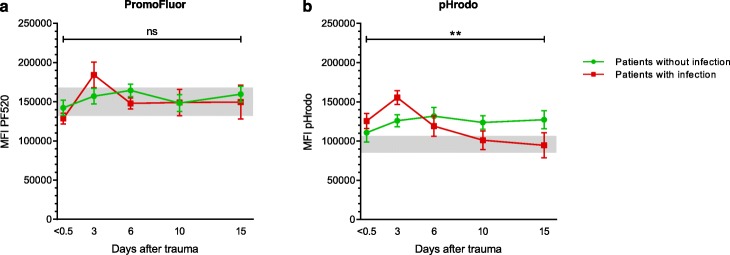
PromoFluor and pHrodo fluorescence. Neutrophil PromoFluor (**a**) and pHrodo (**b**) fluorescence in patients who developed an infection (red square) and patients who did not develop an infection (green circle) after 60 min of incubation with *S. aureus* bioparticles. Patients with infections were compared to patients without infections with generalized estimating equations. Data are presented as mean with the standard error of the mean. Gray areas represent healthy control values (95% confidence interval). MFI = mean fluorescence intensity. PF520 = PromoFluor 520 LSS. *S. aureus = Staphylococcus aureus.* Ns = non-significant. **p < 0.01

Figure 2 depicts neutrophil phagocytosis and neutrophil phagosomal acidification over time after trauma. Neutrophil phagocytosis did not differ between patients and healthy controls (Fig. 2b, c). Also, no statistically significant differences were found in neutrophil phagocytosis over time between patients who developed infectious complications and patients who did not (Fig. 2a, *β* = − 0.041, *p* = 0.928). Neutrophil phagosomal acidification, on the other hand, was significantly different between patients with and without infections (Fig. 2d, *β* = − 0.029, *p* < 0.001). Patients who did not develop infections showed increased acidification 6 days (*p* = 0.029), 10 days (*p* = 0.025), and 15 days (*p* = 0.026) after trauma compared to healthy individuals (Fig. 2e). Patients who developed an infection, however, showed increased acidification immediately after trauma (*p* = 0.006) and after 3 days (*p* = 0.026) compared to healthy individuals, followed by a marked decrease in acidification to low-normal levels 6, 10, and 15 days after trauma (Fig. 2f). When comparing PF520 MFI and pHrodo MFI between outcome groups (Fig. 3a, b), no significant differences were found in PF520 MFI (*β* = − 381, *p* = 0.755), whereas pHrodo MFI significantly differed between outcome groups (*β* = − 3339, *p* = 0.004). No correlation was found between baseline characteristics and acidification (Supplementary Table 1).

## Discussion

The assay on neutrophil acidification as described in this paper showed clear differences in neutrophil phagosomal activity between patients who developed infectious complications and patients who did not. These differences were indicated by differences in pHrodo fluorescence intensity and became more evident when pHrodo fluorescence intensity was divided by PF520 fluorescence intensity to correct for the number of phagocytosed bioparticles per cell, thereby solely investigating acidification. Neutrophil phagosomal acidification was increased within the first 3 days after trauma in patients who later developed infectious complications. Thereafter, neutrophil phagosomal acidification decreased in these patients to levels in the lower normal range. In marked contrast, in neutrophils from patients who did not develop an infection, phagosomal acidification was within the normal range at first, but increased to above reference values during the second week after trauma.

In the past years, many studies focused on neutrophil phagocytosis, but only a limited number of studies investigated the whole process of neutrophil antibacterial function including intracellular processing of bacteria [28, 29, 39]. The whole process of neutrophil antibacterial function was analyzed by measuring bacterial proliferation over hours to days in the presence of isolated neutrophils in suspension or in tissue-like scaffolds [28, 29, 39]. However, these analyses were laborious, time-consuming, and required experienced personnel. Moreover, these analyses required manual isolation of neutrophils which is known to cause artifacts and reduce reproducibility [40, 41]. Therefore, there has been an unmet need for a fast, reproducible, and clinical applicable point-of-care test without the need for (manual) neutrophil isolation. Here we demonstrate a new assay in which neutrophil function can be measured fully automated and point-of-care within 60 min. Moreover, this assay does not only include neutrophil phagocytosis, but also includes neutrophil acidification, as a measurement of intracellular bacterial processing.

It was remarkable that trauma patients who developed infectious complications initially showed better neutrophil acidification than patients who did not. Since different neutrophil subsets exhibit differences in phagosomal acidification (Supplementary Figure 3), an explanation for this finding could have been the presence of better acidifying neutrophils (CD16^dim^/CD62L^bright^) in the peripheral blood of these patients [37]. However, we found no differences in neutrophil subset percentages between these severely injured patients who developed an infection and patients who did not. Hence, the increased acidification was not simply the result of differences in specific neutrophil subsets in the blood.

Next, the increased acidification could be due to neutrophil priming caused by inflammatory mediators or damage-associated molecular patterns (DAMPs) released by the extensive tissue damage and disrupted protective barriers through which microbes could have entered the body [42]. This hypothesis was supported by the increased acidification within the first 3 days that was most evident in patients who later developed an infection. This suggested that these patients exhibited a more pronounced inflammatory response. Since neutrophil lifespan is estimated to be 4–5 days and it takes 6–7 days to mobilize new neutrophils into the blood [43, 44], early mobilization (1–2 days after trauma) of too many well-functioning neutrophils might have led to a relative shortage of such neutrophils after 6 days. This is supported by the decrease in phagosomal acidification observed in patients at the time of infection. Moreover, it is possible that the infections caused a migration of well-functioning neutrophils into tissues leading to a further decrease of well-functioning neutrophils in the blood, a phenomenon previously described as a refractory immune state [45].

Surprisingly, ISS [1, 46] did not significantly differ between patients who developed infections and patients who did not, whereas it has recently been found that both correlated with the risk of infectious complications. Most likely, this was because we only included “high-risk” patients by selecting the most severely injured patients for this study. It should be taken into account that the study population was small. Although the study population was sufficient for the aim of this proof-of-principle study, it is possible that with a larger study population, ISS and resuscitation would have significantly differed between patients with and without infection. However, despite low patient numbers, we still found clear differences in neutrophil phagosomal acidification already before patients developed their infection. This suggests that the neutrophil phagosomal acidification assay is a valuable test for predicting and monitoring high-risk patients for infectious complications after trauma.

For future optimization of this assay, several aspects can be considered. First, although granulocytes and lymphocytes can be easily distinguished on their specific forward/side scatter pattern (Supplementary Figure 1a), it is not always possible to distinguish monocytes. Therefore, if monocyte analysis is warranted, a monocyte-specific marker should be used. Secondly, the positive and negative populations measured by the PF520 signal somewhat overlap. Therefore, it might be valuable to investigate other brighter fluorochromes. An additional advantage of this could be that a brighter fluorochrome might enable better quantification of the number of phagocytosed bioparticles per cell, which could be of interest when investigating the phagocyte capacity of specific cell types. Thirdly, it should be taken into account that temperature changes influence the outcomes of this assay. Although acidification remains relatively stable, phagocytosis clearly increases as the temperature increases (Supplementary Figure 4). To ensure assay stability, it is therefore recommended to perform this analysis in a room with a constant temperature. Lastly, our gating strategy was based on visual estimation by 2 independent researchers. For future studies, it is worthwhile to investigate a more standardized gating strategy.

## Conclusion

Neutrophil function in terms of phagocytosis and acidification can now be measured quickly and fully automated in the ICU as point-of-care test. After severe trauma, neutrophil phagosomal acidification differs between patients who develop infectious complications and patients who do not. Hence, this new assay might be an asset to monitor the inflammatory status of trauma patients in the ICU and identify patients who develop infectious complications.

## Supplementary information

**Additional file 1: Supplementary Figure 1.** Gating strategy for the determination of neutrophil phagocytosis and acidification. Granulocytes and lymphocytes can be distinguished on the forward scatter (FS)/side scatter (SS) **(a)**. Neutrophils were identified by selecting granulocytes with CD16 expression (thereby excluding eosinophils) **(b)**. Combined analysis of pHrodo® Green fluorescence and PF520 fluorescence allows for assessment of phagocytosis, expressed as a percentage of PF520-positive neutrophils **(c)**, and neutrophil phagosomal acidification, expressed as the ratio pHrodo® Green fluorescence divided by PF520 fluorescence **(d)**. PF520 = PromoFluor 520 LSS.

**Additional file 2: Supplementary Figure 2.** Gating strategy for distinguishing neutrophil subsets. The gating of the neutrophil subsets CD16^dim^/CD62L^bright^, CD16^bright^/CD62L^bright^ and CD16^bright^/ CD62L^dim^ is shown.

**Additional file 3: Supplementary Table 1.** Correlation between baseline characteristics and acidification. The relation between acidification and baseline variables was analyzed. For continuous variables (age, ISS, RBCs, FFPs and PLTs), correlation was analyzed using the Spearman’s rho test because data were not normally distributed. Correlation coefficient and p-value are reported. No statistically significant correlations were found. The relation between acidification and gender was analyzed using a Mann-Whitney U test, because data were not normally distributed. U-value and p-value are reported. No statistically significant differences were found.

**Additional file 4: Supplementary Figure 3.** Neutrophil phagosomal acidification per neutrophil subset. Neutrophil phagosomal acidification of CD16^dim^/CD62L^bright^ cells (), CD16^bright^/CD62L^bright^ cells () and CD16^bright^/ CD62L^dim^ cells () in all patients after 10, 20, 40 and 60 minutes of incubation with *S. Aureus* bioparticles. Neutrophil phagosomal acidification after 60 minutes was compared between subsets using a one-way ANOVA. Significant differences were found between subsets (p < 0.001). A follow-up comparison of the means was performed with a Tukey’s correction for multiple comparisons. CD16dim/CD62Lbright neutrophils were found to acidify significantly better than CD16^bright^/CD62Lbright neutrophils (p = 0.016) and then CD16^bright^/CD62L^bright^ neutrophils (p < 0.001). MFI = median fluorescence intensity. PF520 = PromoFluor 520 LSS. *S. Aureus = Staphylococcus Aureus.* Data are presented as mean with standard error of the mean. *P<0.05, **P<0.01, ***P<0.001.

**Additional file 5: Supplementary Figure 4.** Effect of temperature changes on neutrophil function. Neutrophil phagocytosis **(a)** and neutrophil phagosomal acidification **(b)** in five healthy controls at different temperatures. Blood from 5 healthy controls was analyzed after incubation for 60 minutes with double-labeled bioparticles on ice, in a water bath of 25°C and in a water bath of 37°C. Then, red blood cells were lysed using lysing reagent A and lysing reagent B from the AQUIOS CL® “Load & Go” flow cytometer and leukocyte analysis was performed using the BD FACSCanto™ II (BD Biosciences). Temperature conditions were compared using a Friedman test and a Mann-Whitney *U* Test with a Dunn's correction for multiple comparisons. Neutrophil phagocytosis and neutrophil acidification significantly differed at different temperatures (p = 0.0008 and p = 0.0394, respectively). Neutrophil phagocytosis increased as the temperature increased, and significant differences were found between samples that were kept on ice and samples that were kept in 37°C (p = 0.005). Such a temperature dependent trend was not observed for neutrophil acidification. However, significant differences were found between 25°C and 37°C (p = 0.034).

**Additional file 6: Supplementary Figure 5.** Effect of CD16/CD62L-antibodies on neutrophil function. Neutrophil phagocytosis (**a**) and neutrophil phagosomal acidification (**b**) in five healthy controls after 60 minutes of incubation with *S. Aureus* bioparticles. Per patient, this analysis was performed three times in different conditions: 1) with 6 μL Hepes buffer, 2) with 6 μL CD16-BV785 and 3) with 6 μL CD62L-BV650. The Hepes buffer and antibody-fluorochrome combinations were added to the wells plate prior to initiation of the functional analyses to prevent a time delay between the different analyses. The Hepes buffer consisted of 20 mM Hepes, 132 mM NaCl, 6 mM KCl, 1.2 mM KH_2_PO_4_ and 1 mM MgSO_4_, supplemented with 5 mM glucose, 1 mM CaCl_2_, and 0.5% (w/v) human serum albumin. The CD16/CD62L-antibody-fluorochrome combinations were chosen because the fluorochromes are not excited by the 488 laser of the AQUIOS CL® “Load & Go” flow cytometer. A Friedman test was used to compare the three different conditions. No significant differences in neutrophil phagocytosis and neutrophil phagosomal acidification were found after the addition of CD16 and CD62L antibodies. Data are presented as individual values with mean (black line). MFI = median fluorescence intensity. PF520 = PromoFluor 520 LSS. *S. Aureus = Staphylococcus Aureus.* Ns = non-significant.

**Additional file 7: Supplementary Figure 6.** Bioparticles were internalized in neutrophils. ImageStream analysis showed that the bioparticles (green) were internalized in the neutrophil (staining of CD16 on cell membrane in blue). The experiment was performed using the Amnis® ImageStream®XMk II and data were analyzed using Exploration Software (IDEAS, Luminex, Austin, USA).

## Data Availability

The datasets used and/or analyzed during the current study are available from the corresponding author on reasonable request.

## Abbreviations

AIS: Abbreviated injury scale
CRP: C-reactive protein
DAMPs: Damage-associated molecular patterns
fMLF: Formyl-methionyl-leucyl-phenylalanine
GEE: Generalized estimating equations
ICU: Intensive care unit
IQR: Interquartile range
ISS: Injury severity score
MFI: Mean fluorescence intensity
Ns: Non-significant
PF520: PromoFluor 520 LSS
RBC: Red blood cells
*S. aureus*: *Staphylococcus aureus*
