# Supplementary figures and images for: New automated analysis to monitor neutrophil function point-of-care in the intensive care unit after trauma
Source: Intensive Care Med Exp. 2020 Mar 14;8:12. doi: 10.1186/s40635-020-0299-1 (PMC7072076; doi:10.1186/s40635-020-0299-1)

**a**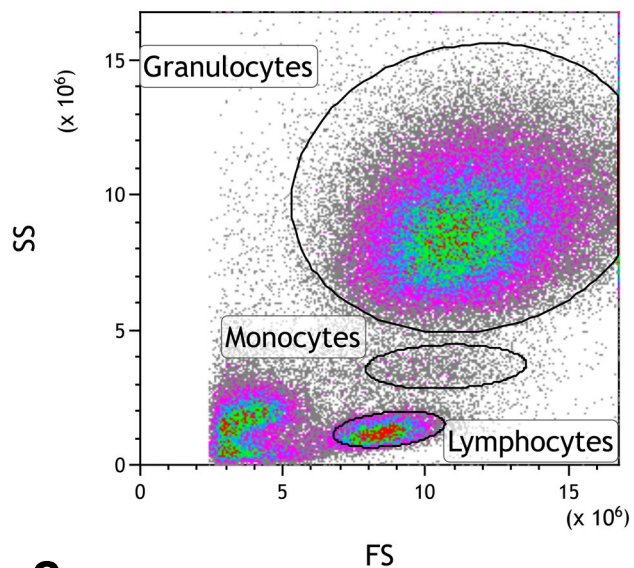**b**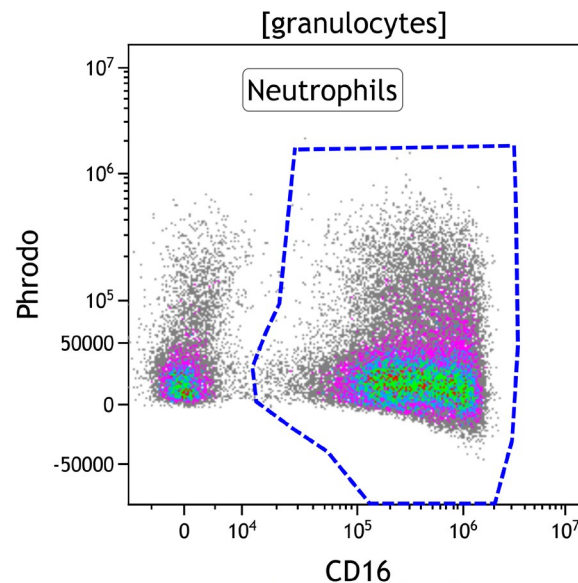**c**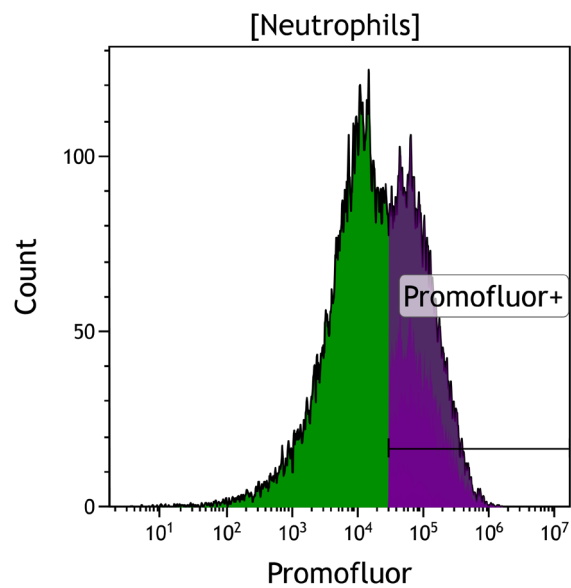**d**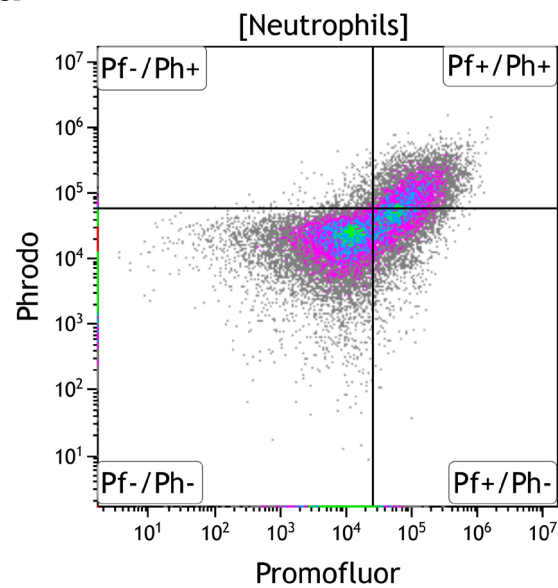

Supplement: Supplementary file 1 — Additional file 1: Supplementary Figure 1. Gating strategy for the determination of neutrophil phagocytosis and acidification. Granulocytes and lymphocytes can be distinguished on the forward scatter (FS)/side scatter (SS) (a). Neutrophils were identified by selecting granulocytes with CD16 expression (thereby excluding eosinophils) (b). Combined analysis of pHrodo® Green fluorescence and PF520 fluorescence allows for assessment of phagocytosis, expressed as a percentage of PF520-positive neutrophils (c), and neutrophil phagosomal acidification, expressed as the ratio pHrodo® Green fluorescence divided by PF520 fluorescence (d). PF520 = PromoFluor 520 LSS. [file 40635_2020_299_MOESM1_ESM.pdf]

**a**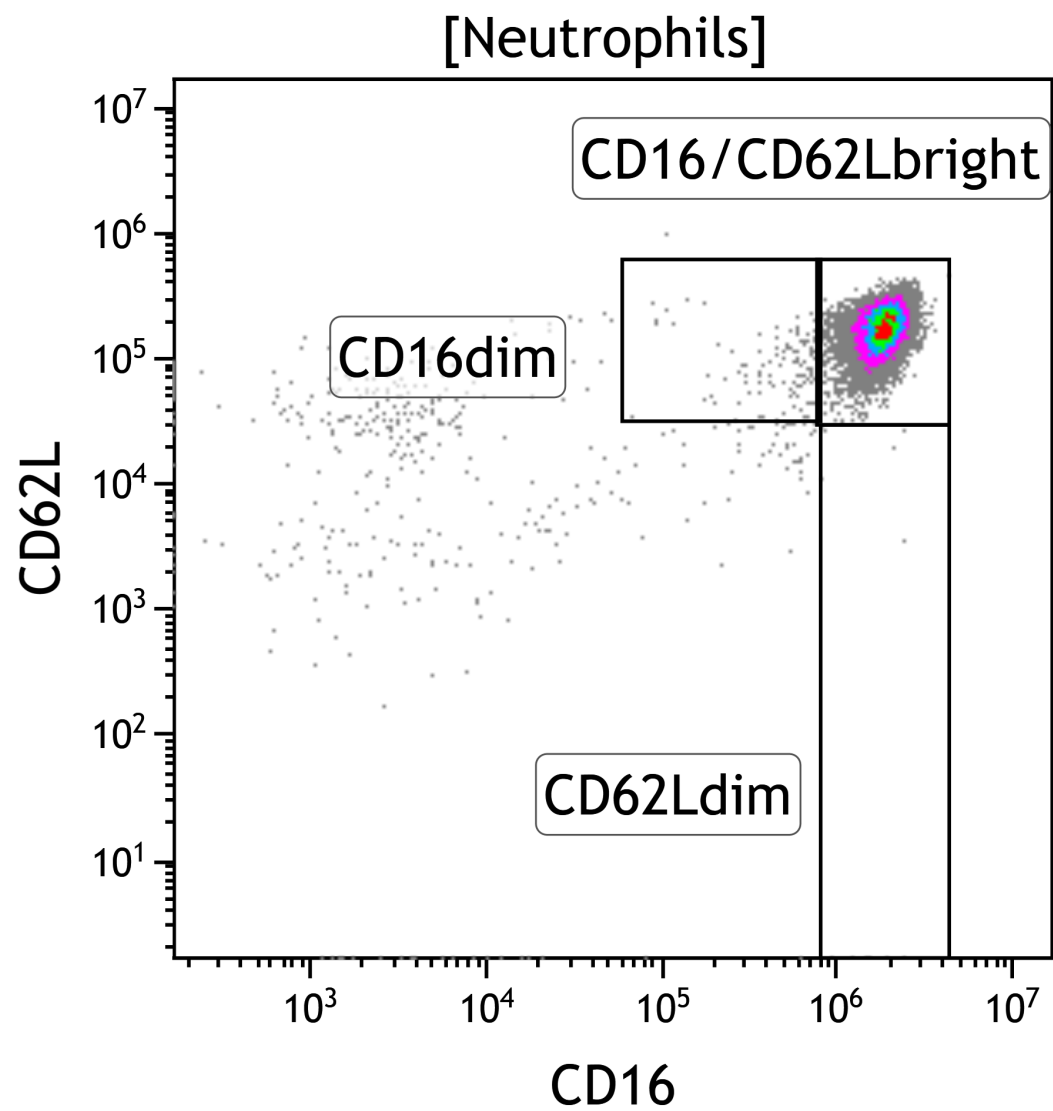**b**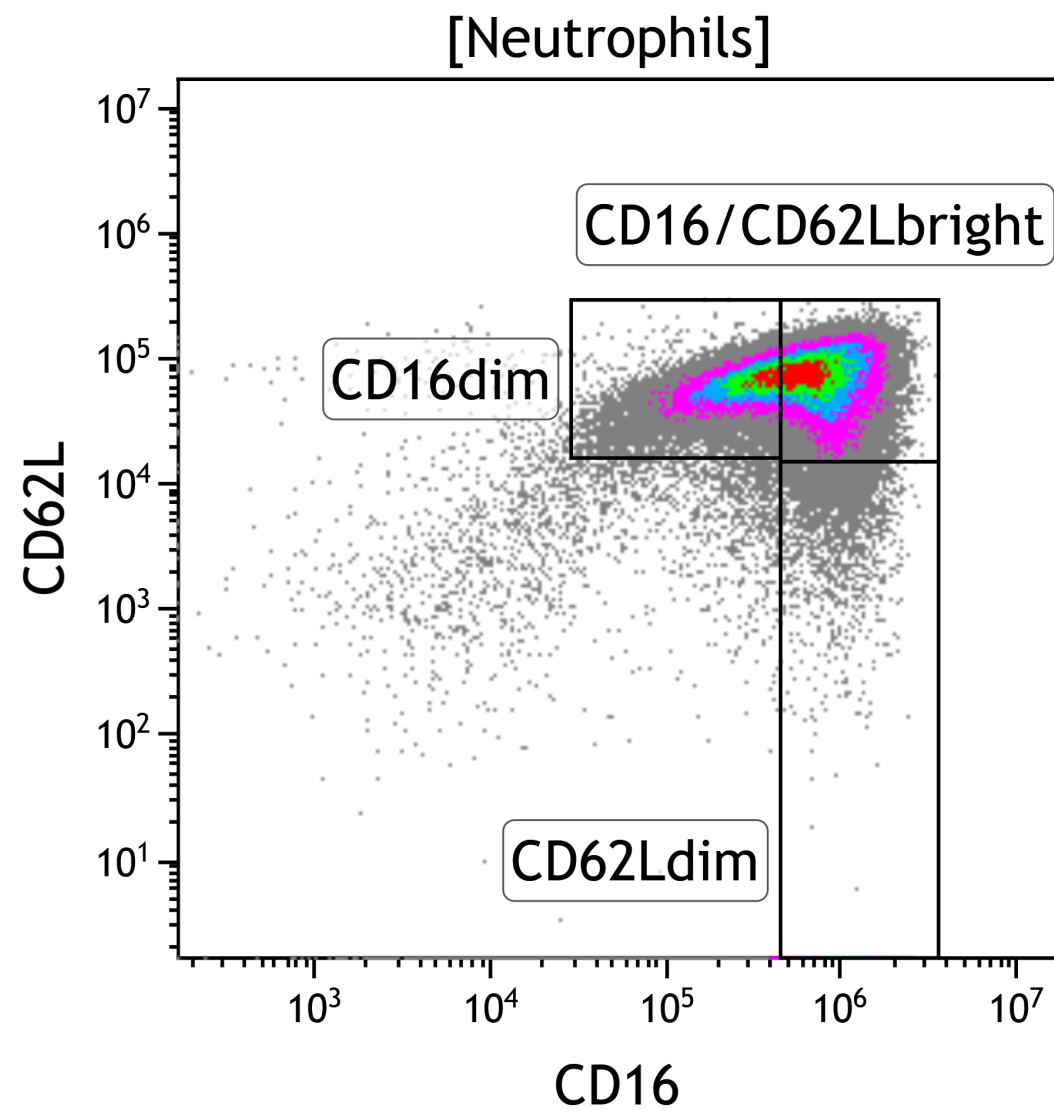

Supplement: Supplementary file 2 — Additional file 2: Supplementary Figure 2. Gating strategy for distinguishing neutrophil subsets. The gating of the neutrophil subsets CD16dim/CD62Lbright, CD16bright/CD62Lbright and CD16bright/ CD62Ldim is shown. [file 40635_2020_299_MOESM2_ESM.pdf]

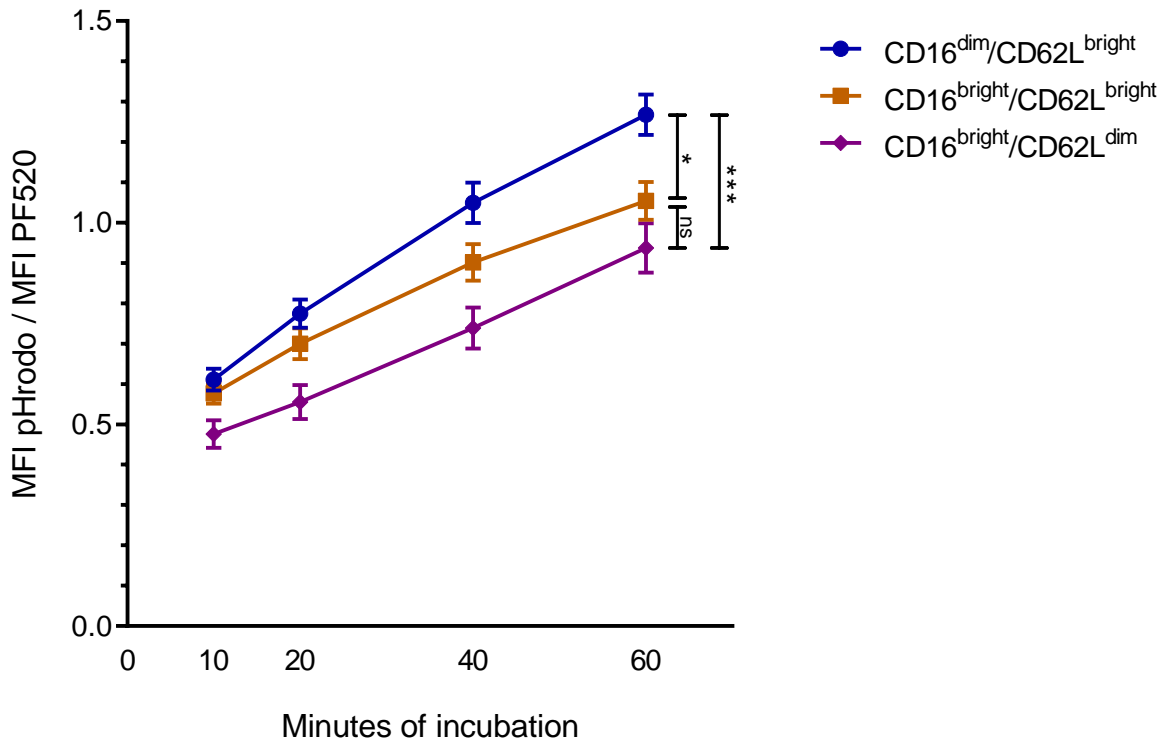

Supplement: Supplementary file 4 — Additional file 4: Supplementary Figure 3. Neutrophil phagosomal acidification per neutrophil subset. Neutrophil phagosomal acidification of CD16dim/CD62Lbright cells (), CD16bright/CD62Lbright cells () and CD16bright/ CD62Ldim cells () in all patients after 10, 20, 40 and 60 minutes of incubation with S. Aureus bioparticles. Neutrophil phagosomal acidification after 60 minutes was compared between subsets using a one-way ANOVA. Significant differences were found between subsets (p < 0.001). A follow-up comparison of the means was performed with a Tukey’s correction for multiple comparisons. CD16dim/CD62Lbright neutrophils were found to acidify significantly better than CD16bright/CD62Lbright neutrophils (p = 0.016) and then CD16bright/CD62Lbright neutrophils (p < 0.001). MFI = median fluorescence intensity. PF520 = PromoFluor 520 LSS. S. Aureus = Staphylococcus Aureus. Data are presented as mean with standard error of the mean. *P<0.05, **P<0.01, ***P<0.001. [file 40635_2020_299_MOESM4_ESM.pdf]

**a****Phagocytosis**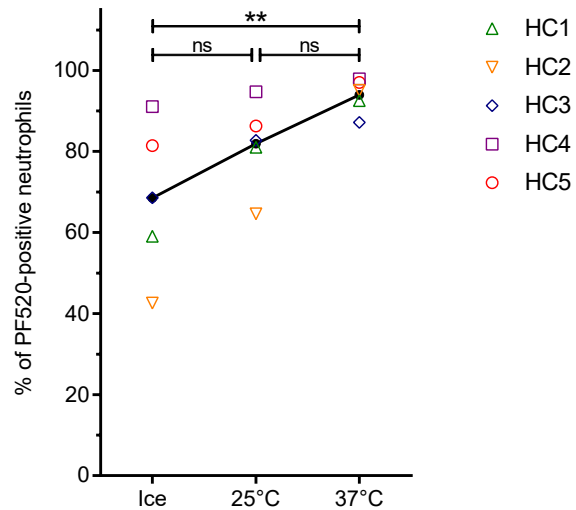**b****Acidification**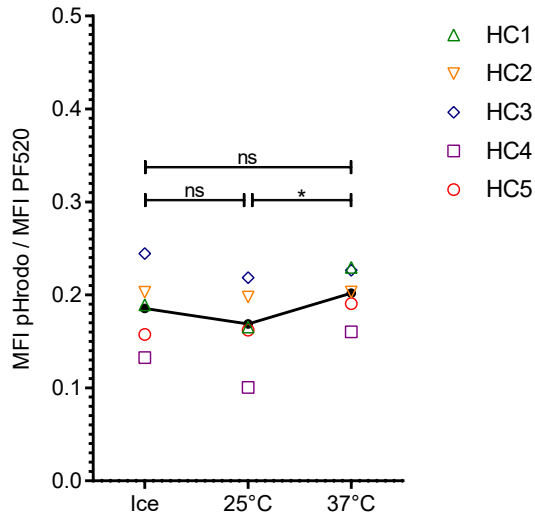

Supplement: Supplementary file 5 — Additional file 5: Supplementary Figure 4. Effect of temperature changes on neutrophil function. Neutrophil phagocytosis (a) and neutrophil phagosomal acidification (b) in five healthy controls at different temperatures. Blood from 5 healthy controls was analyzed after incubation for 60 minutes with double-labeled bioparticles on ice, in a water bath of 25°C and in a water bath of 37°C. Then, red blood cells were lysed using lysing reagent A and lysing reagent B from the AQUIOS CL® “Load & Go” flow cytometer and leukocyte analysis was performed using the BD FACSCanto™ II (BD Biosciences). Temperature conditions were compared using a Friedman test and a Mann-Whitney U Test with a Dunn's correction for multiple comparisons. Neutrophil phagocytosis and neutrophil acidification significantly differed at different temperatures (p = 0.0008 and p = 0.0394, respectively). Neutrophil phagocytosis increased as the temperature increased, and significant differences were found between samples that were kept on ice and samples that were kept in 37°C (p = 0.005). Such a temperature dependent trend was not observed for neutrophil acidification. However, significant differences were found between 25°C and 37°C (p = 0.034). [file 40635_2020_299_MOESM5_ESM.pdf]

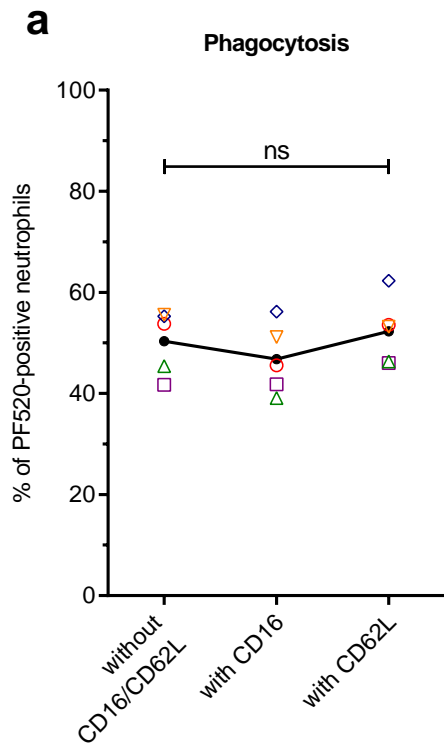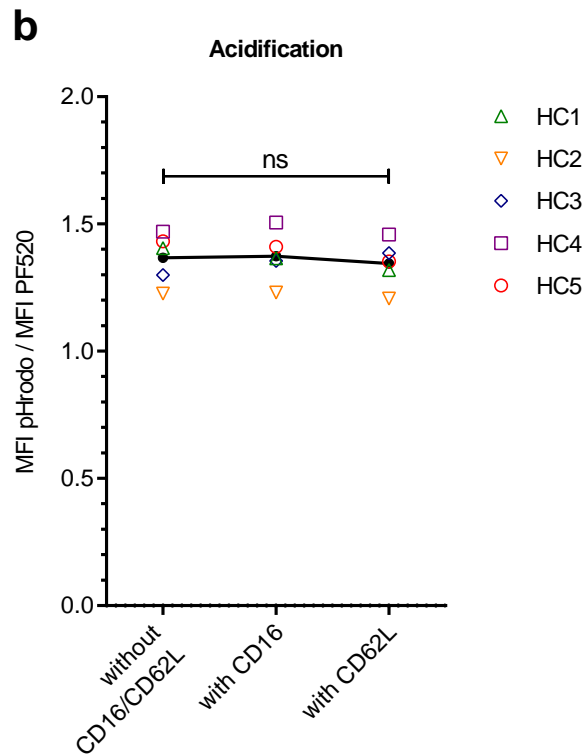

Supplement: Supplementary file 6 — Additional file 6: Supplementary Figure 5. Effect of CD16/CD62L-antibodies on neutrophil function. Neutrophil phagocytosis (a) and neutrophil phagosomal acidification (b) in five healthy controls after 60 minutes of incubation with S. Aureus bioparticles. Per patient, this analysis was performed three times in different conditions: 1) with 6 μL Hepes buffer, 2) with 6 μL CD16-BV785 and 3) with 6 μL CD62L-BV650. The Hepes buffer and antibody-fluorochrome combinations were added to the wells plate prior to initiation of the functional analyses to prevent a time delay between the different analyses. The Hepes buffer consisted of 20 mM Hepes, 132 mM NaCl, 6 mM KCl, 1.2 mM KH2PO4 and 1 mM MgSO4, supplemented with 5 mM glucose, 1 mM CaCl2, and 0.5% (w/v) human serum albumin. The CD16/CD62L-antibody-fluorochrome combinations were chosen because the fluorochromes are not excited by the 488 laser of the AQUIOS CL® “Load & Go” flow cytometer. A Friedman test was used to compare the three different conditions. No significant differences in neutrophil phagocytosis and neutrophil phagosomal acidification were found after the addition of CD16 and CD62L antibodies. Data are presented as individual values with mean (black line). MFI = median fluorescence intensity. PF520 = PromoFluor 520 LSS. S. Aureus = Staphylococcus Aureus. Ns = non-significant. [file 40635_2020_299_MOESM6_ESM.pdf]

Ch02/Ch03/Ch04

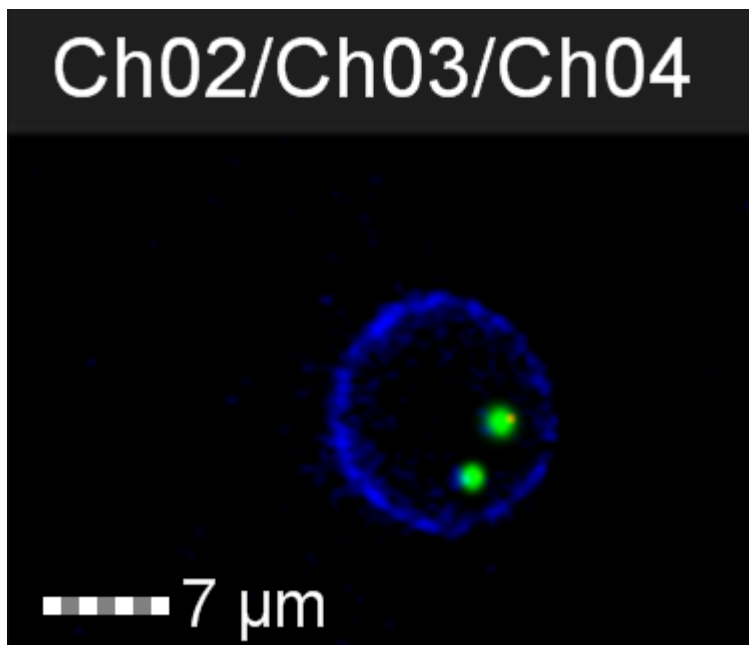

Supplement: Supplementary file 7 — Additional file 7: Supplementary Figure 6. Bioparticles were internalized in neutrophils. ImageStream analysis showed that the bioparticles (green) were internalized in the neutrophil (staining of CD16 on cell membrane in blue). The experiment was performed using the Amnis® ImageStream®XMk II and data were analyzed using Exploration Software (IDEAS, Luminex, Austin, USA). [file 40635_2020_299_MOESM7_ESM.pdf]
