# Supplementary material for: New automated analysis to monitor neutrophil function point-of-care in the intensive care unit after trauma
Source: Intensive Care Med Exp. 2020 Mar 14;8:12. doi: 10.1186/s40635-020-0299-1 (PMC7072076; doi:10.1186/s40635-020-0299-1)
Supplement: Supplementary file 3 — Additional file 3: Supplementary Table 1. Correlation between baseline characteristics and acidification. The relation between acidification and baseline variables was analyzed. For continuous variables (age, ISS, RBCs, FFPs and PLTs), correlation was analyzed using the Spearman’s rho test because data were not normally distributed. Correlation coefficient and p-value are reported. No statistically significant correlations were found. The relation between acidification and gender was analyzed using a Mann-Whitney U test, because data were not normally distributed. U-value and p-value are reported. No statistically significant differences were found. [file 40635_2020_299_MOESM3_ESM.pdf]

	Acidification <12 h	Acidification day 3	Acidification day 6	Acidification day 10	Acidification day 15
Age	r = -0.516, *p* = 0.059	r = 0.042, *p* = 0.887	r = -0.130, *p* = 0.659	r = -0.346, *p* = 0.247	r = -0.383, *p* = 0.308
Gender	*U* = 8.0, *p* = 0.106	*U* = 18.0, *p* = 0.606	*U* = 18.0, *p* = 0.839	*U* = 18.0, *p* = 1.000	*U* = 6.0, *p* = 0.889
ISS	r = -0.102, *p* = 0.729	r = 0.269, *p* = 0.325	r = 0.219, *p* = 0.452	r = 0.083, *p* = 0.788	r = -0.370, *p* = 0.327
RBCs	r = -0.016, *p* = 0.957	r = 0.436, *p* = 0.119	r = 0.029, *p* = 0.921	r = -0.095, *p* = 0.758	r = -0.443, *p* = 0.233
FFP	r = 0.258, *p* = 0.372	r = 0.400, *p* = 0.156	r = 0.236, *p* = 0.417	r = -0.119, *p* = 0.699	r = -0.070, *p* = 0.859
PLTs	r = 0.220, *p* = 0.449	r = 0.390, *p* = 0.168	r = -0.074, *p* = 0.801	r = -0.228, *p* = 0.453	r = -0.183, *p* = 0.638

ISS = Injury Severity Score. FFP = fresh frozen plasma. RBCs = packed red blood cells. PLTs = platelets.
